# Morphology and Prevalence of the Inferior Transverse Scapular Ligament: Systematic Review, Meta-Analysis, and Proposal for Classification

**DOI:** 10.3390/medicina60091504

**Published:** 2024-09-14

**Authors:** Ioannis Antonopoulos, Evmorfia Pechlivanidou, Łukasz Hubert Olewnik, Nicol Zielinska, Dimosthenis Chrysikos, Alexandros Samolis, George Tsikouris, Theodore Troupis

**Affiliations:** 1 Department of Anatomy, School of Medicine, Faculty of Health Sciences, National and Kapodistrian University of Athens, 11527 Athens, Greece; ikantonopoulos@gmail.com (I.A.);; 2 1st Department of Orthopaedics, General Children’s Hospital of Athens “P. & A. Kyriakou”, 11527 Athens, Greece; evmorfia.pechlivanidou@gmail.com; 3 Department of Hygiene, Epidemiology and Medical Statistics, School of Medicine, Faculty of Health Sciences, National and Kapodistrian University of Athens, 11527 Athens, Greece; 4 Department of Anatomical Dissection and Donation, Lodz Medical University, 90-419 Lodz, Poland

**Keywords:** spinoglenoid ligament, shoulder anatomy, morphological subtypes, inferior transverse scapular ligament, suprascapular nerve, classification system

## Abstract

*Background/Objectives*: The suprascapular nerve is most vulnerable to entrapment at the suprascapular and spinoglenoid notches, causing neuropathy. Numerous studies have examined the suprascapular notch and ligament and its relationship with suprascapular nerve entrapment, but few have examined the spinoglenoid notch and the inferior transverse scapular ligament (ITSL). This study summarizes all existing ITSL morphology studies and presents a simple and comprehensive classification system for different ITSL subtypes. *Methods:* A systematic review of the literature was conducted according to the PRISMA guidelines, searching the online databases PubMed and Embase. The references of each relevant article were further screened to find more eligible studies. The Anatomical Quality Assessment tool was used in order to further evaluate the quality of the records extracted. STATA MP 14 was used for the analysis in this study. *Results*: In total, 14 studies (995 scapulae; minimum: 1 and maximum: 268) were included in the present study. The overall ITSL prevalence was 5.8 (95% CI: 4.5–7.1) and the estimated odds for ligamentous vs. membranous type was 0.5 (95% CI: 0.3–0.7). The basic different morphological subtypes of the ITSL reported in the included studies are the band-like ligament, the fan-shaped ligament, the membranous ITSL, and the perforated membranous types. *Conclusions:* The ITSL represents an anatomical structure of mostly ligamentous nature. A single ITSL definition and standardization of its basic morphological subtypes along with an easy-to-remember and thus widely used classification system could greatly facilitate the comprehensive description, identification, and proper handling of this element across many surgical procedures.

## 1. Introduction

The suprascapular nerve (SSN) is a mixed nerve originating from the superior trunk of the brachial plexus (C4–C6 spinal nerves) [1,2]. Its motor component innervates the supraspinatus and infraspinatus muscles, while its sensory component supplies the acromioclavicular and glenohumeral joints and is responsible for the proprioception of the supraspinatus and infraspinatus muscles [3,4]. Lateral and medial subacromial branches emerging from it have also been reported in the literature [3,5,6]. The SSN follows a course through the posterior triangle of the neck, underneath the trapezius and parallel to the inferior belly of the omohyoid muscle. It enters the supraspinous fossa through the suprascapular notch, running under the superior transverse scapular ligament, and then passes deep to the supraspinatus muscle [7]. Finally, the SSN reaches the infraspinous fossa after passing through the incisura spinoglenoidea (or “spinoglenoid notch”), which is bridged by the inferior transverse scapular ligament (ITSL), the so-called “spinoglenoid ligament” [7].

The spinoglenoid notch is the second most common point along the course of the SSN (after the suprascapular notch) where an injury or entrapment can occur [8], which can potentially result in suprascapular neuropathy [9,10]. This could be the result of a hypertrophic or ossified ITSL [11,12,13], a ganglion cyst originating either from the subacromial bursa [14] or posterior capsule of the shoulder joint [15], or even enlarged spinoglenoid veins [16].

Although there is a considerable number of studies in the literature describing the anatomical and morphometrical features of the suprascapular notch and ligament [10,17,18,19,20], as well as their correlation with SSN entrapment, only a few similar studies about the spinoglenoid notch and ITSL exist [12,21,22,23,24,25,26,27,28,29,30,31,32,33].

The aim of this study is to summarize and compare, for the first time, all the existing studies concerning the ITSL morphology in a comprehensive way, thus filling this gap in the literature. In addition, we propose a concise and easy-to-remember classification system of different ITSL types based on the main findings of these studies.

## 2. Materials and Methods

The authors designed this systematic review and meta-analysis according to the Preferred Reporting Items for Systematic Reviews and Meta-Analyses (PRISMA) [34].

### 2.1. Literature Search

A systematic review of the literature was conducted, searching the online databases PubMed and Embase, from 1975 until 6 May 2023. The search terms used were as follows: “spinoglenoid ligament”, “inferior transverse scapular ligament”, “spinoglenoid ligament morphology”, “spinoglenoid ligament anatomy”, “inferior transverse scapular ligament anatomy”, and “inferior transverse scapular ligament morphology”. The above-mentioned terms were used with all possible combinations for the final results of the search to be extracted. The references of each relevant article were further screened to find more eligible studies (the so-called “snowballing technique”) [35].

### 2.2. Study Selection

Three authors (I.A., E.P., and L.H.O.) screened and selected the articles individually. Original studies and case reports, written in English, German, or French language, concerning the morphology of the ITSL were considered. Since the primary objective of this study was the description and presentation of the morphological types of the ITSL rather than solely focusing on its prevalence, radiological studies were excluded, and only cadaveric studies or case reports were included. Reviews and conference abstracts were excluded. The authors imposed no limitations regarding the demographic characteristics of the specimens (i.e., age, sex, and origin). Any disagreements that emerged between the three authors throughout these processes were settled by unanimous agreement of all authors.

### 2.3. Quality Assessment

The Anatomical Quality Assessment (AQUA) tool was used in order to further evaluate the quality of the records extracted. The AQUA tool was designed to assess the risk of bias of anatomical studies through a five-domain evaluation (Objectives and Subject Characterization, Study Design, Methodology Characterization, Descriptive Anatomy, and Reporting of Results). In every domain, each question can be responded to with either a “yes”, “no”, or “unclear”. If all of the questions pertaining to a specific domain are answered affirmatively, the assessment of bias for that domain is determined to be “low”. A question that has a rating of “no” or “unclear” is considered to have a “high” risk of bias within the domain [36]. Each study was assessed as having a “high” risk of bias if it had 4 or 5 domains with high risk, a “moderate” risk if it had 2 or 3 domains with high risk of bias, or a “low” risk if it had 0–1 domain with high risk. The inclusion criteria for the present study were limited to those articles that were assessed to have a “low” or “moderate” risk of bias.

### 2.4. Data Extraction

A data extraction form was used to identify and record the following data: year and country where the study was conducted, type of the study (case series or case report), number of specimens used, type of the specimens (embalmed cadavers, fresh frozen cadavers, or dried bones), as well as the reported prevalence of the ITSL, its nature, and its morphology.

### 2.5. Statistical Analysis

The data extracted from the eligible studies included the total number of examined specimens and the corresponding number of ITSLs. Additionally, for the examined specimens, the total number of ligamentous, membranous, and ossified ITSLs was extracted and recorded. For the examined specimens, the origin and the site of ITSL insertion as well as the length of its upper and lower margin was recorded. The characteristics of the eligible studies, including their type (i.e., dried bones, fresh frozen specimens, computed tomography (CT) studies, and their combinations), were also recorded in the database. All the data were initially entered into Microsoft Excel (Redmond, WA, USA) and then transferred to Stata, version 14 (StataCorp, College Station, TX, USA), for further analysis.

To assess the prevalence of the studied characteristics, we estimated the relative risk for a person to have the structure. Thus, the ITSL prevalence was estimated as the ratio of the presence of the characteristic and its absence (i.e., ratio = (characteristic/n-characteristic)), derived using meta-analysis, in which the above-mentioned ratio was treated as the effect size. This approach was decided due to the fact that cadaveric studies are not random or representative sample studies to assess the real prevalence in the population; thus, it is epidemiologically more valid to assess the prevalence via this ratio [37,38]. The same methodology was used to assess the prevalence of each type of ITSL (i.e., ligamentous, membranous, and osseous). For the small number of studies recording different types of ITSL, a fair comparison of the odds of ligamentous vs. membranous type to derive the estimated odds after standard meta-analysis was performed. Additionally, regarding the ITSL insertion, the odds for posterior glenoidal neck vs. posterior shoulder capsule were estimated via standard meta-analysis.

The researchers evaluated the heterogeneity of the papers included in the analysis using the Chi-square test and the I^2^ statistic. The Cochran’s Q *p*-value, which was found to be less than 0.10 in the Chi-square test, suggested the presence of significant heterogeneity among the studies. The I^2^ statistic was interpreted using the following intervals. According to the source cited, a range of 0–40% may suggest that a certain factor “might not be important”. In the range of 30–60%, it is suggested that this “might indicate moderate heterogeneity”. For the range of 50–90%, it is said that this “may indicate substantial heterogeneity”. Lastly, in the range of 75–100%, it is mentioned that this “may represent considerable heterogeneity” [39,40].

To determine the origins of variability, subgroup analysis was conducted based on many factors, such as the type of study and geographical region (continent and country) using the respective ratios. In order to further investigate the underlying causes of variation, a sensitivity analysis was conducted on studies that had a sample size of 50 people or more, as deemed appropriate. Statistical analysis was used to evaluate confidence intervals among the groups in order to ascertain the presence of significant differences. The observed overlap within the confidence intervals indicated that the observed differences between the groups were not statistically significant.

### 2.6. Compliance with Ethics Guidelines

This article presents a review of previously conducted studies, in accordance with the PRISMA guidelines.

## 3. Results

### 3.1. Search Results

**Figure 1:**
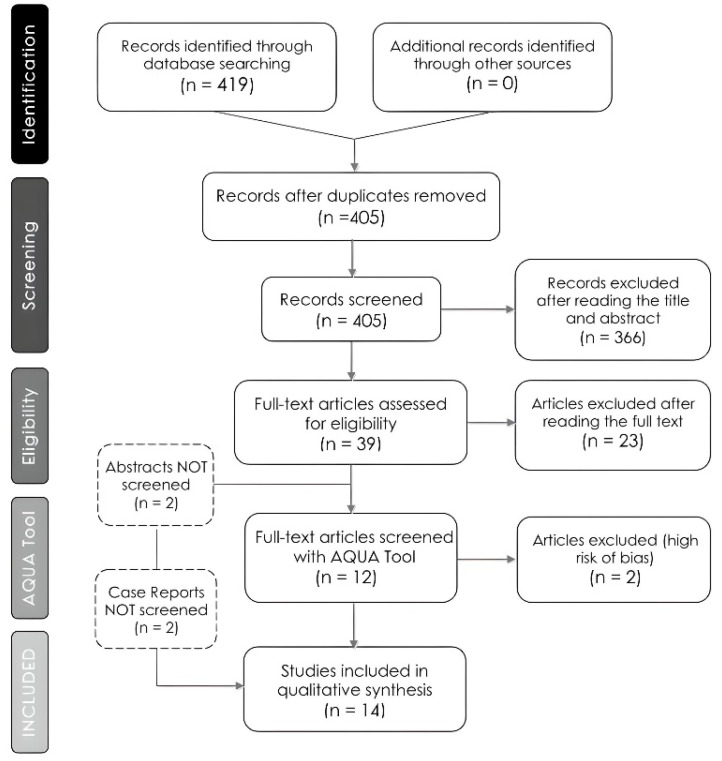
The PRISMA flowchart of our literature search.

The primary literature search yielded a total of 419 papers, out of which 405 were deemed ineligible for inclusion. As a result, 14 [12,21,22,23,24,25,26,27,28,29,30,31,32,33] studies (995 scapulae; minimum: 1 and maximum: 268) in total were included in the present study. The flowchart of the search strategy we followed is schematically depicted in Figure 1.

It is worth noting that 12 [21,22,23,24,25,26,27,28,30,31,32,33] out of the total 14 [12,21,22,23,24,25,26,27,28,29,30,31,32,33] studies were case series, while 2 [12,29] were case reports, which the authors unanimously agreed were of a high level of significance and should thus be included in the present review. In addition, 2 [25,27] of the 12 [21,22,23,24,25,26,27,28,30,31,32,33] original studies were published only as abstracts, though they provided enough useful information in order to be eligible for this systematic review and, as a result, the authors agreed to include them. The basic characteristics of each study included are provided in Table 1.

### 3.2. Quality Assessment

As a last step of the eligibility assessment, we assessed the quality of 12 out of 16 studies we had identified as eligible, using the AQUA Tool. These 12 studies were original anatomical case series published as a full text. Two case reports and two case series that were published as abstracts were not screened via the AQUA Tool. From the 12 studies screened, 2 (16.7%) were excluded, as they were of high risk of bias in 4 out of 5 domains of the AQUA tool. From the other 10 studies, 7 (58.3) were of moderate risk of bias and 3 (25%) were of low risk.

### 3.3. ITSL Prevalence

**Figure 2:**
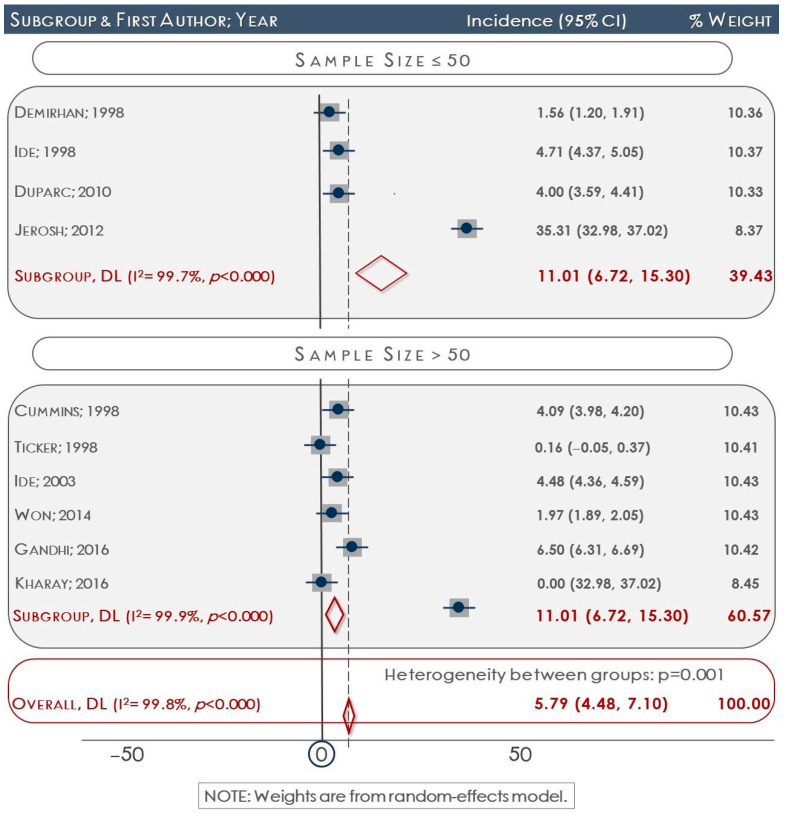
Forest plot regarding sensitivity analyses for the ITSL presence ratio according to studies’ sample size.

The prevalence of the ITSL in each study is shown in Table 2. Based on the data, the overall prevalence was estimated at 5.8 (95% confidence interval (95% CI): 4.5–7.1). The subgroup analysis demonstrated a variation in the ITSL prevalence across different regions. The estimated ratios for the United States, Europe, and Asia were 1.9, 19.5, and 3.7, respectively (*p* < 0.01). The results of sensitivity analyses (*p* < 0.01) indicated that studies with a sample size larger than 50 scapulae provided more accurate estimations of the prevalence of the ITSL compared to studies with a sample size smaller than 50, which overestimated the prevalence (Figure 2) [21,22,24,25,26,27,28,30,32,33].

### 3.4. ITSL Morphology

The basic different morphological subtypes of the ITSL reported in the included studies were the band-like (either quadrangular or hair-like) ligament [21,25,26,28,31,33], the fan-shaped (triangular) ligament [25,33], the membranous ITSL [21,26,28,33], and the perforated (irregular-ethmoid) membranous types [28,33].

**Figure 3:**
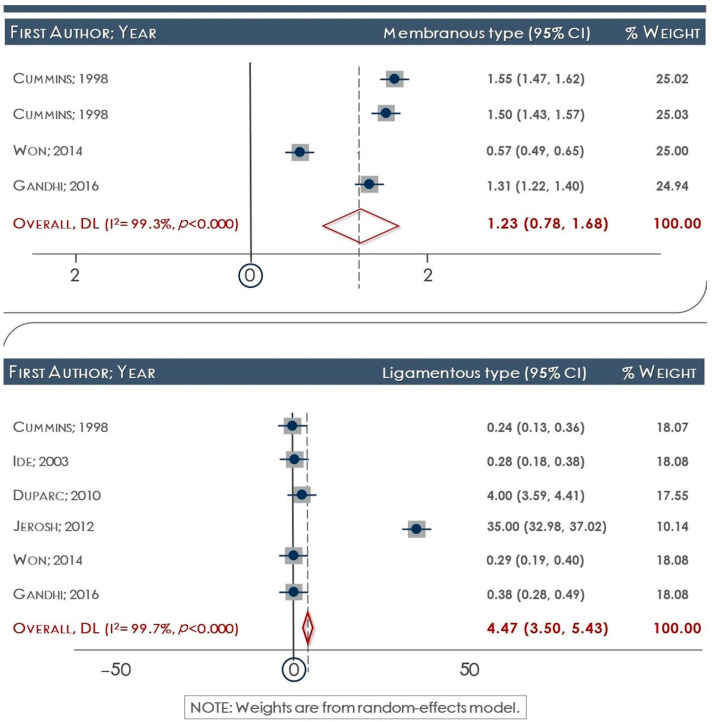
Forest plots reporting the ratio of the membranous (**above**) and ligamentous (**below**) types of the ITSL.

The presence of the ligamentous type was estimated at 4.5 (95% CI: 3.5–5.4), whereas that of the membrane type at 1.2 (95% CI: 0.8–1.6). However, upon analysis of studies that reported both types of the ligament, it was shown that the estimated odds of the ligamentous type vs. the membranous type was 0.5 (95% CI: 0.3–0.7; Figure 3). All of the studies included in this analysis had a sample size exceeding 50 scapulae; hence, a sensitivity analysis was not conducted. In the study conducted by Won et al. (2014) [33], a mixed type was identified at a frequency of 7.3%. In relation to the ossified ligament, it is important to note that only three studies specifically examined this particular trait. It is worth mentioning that two of these studies were case reports, which limited the ability to conduct a meta-analysis on this aspect [21,24,25,26,28,33].

Consensus across several studies indicated that the scapular spine serves as the primary place of origin for the ITSL [12,21,22,23,30,31,32,33]. In terms of their insertion, five studies agreed that all ITSLs were inserted by the posterior glenoidal neck (also referred to as “margin”), whereas one study reported only the posterior shoulder capsule as the site of insertion (Table 3). The estimated odds ratio for the posterior glenoidal neck vs. posterior shoulder capsule was 4.4 (95% CI: 1.3–7.5).

### 3.5. ITSL Morphometrics

Three studies reported the average length of the ITSL, including both the higher and lower mean values. The mean value obtained from the poll was estimated to be 15.5 mm (95% CI: 13.9 mm–17.2 mm) for the higher limit. Similarly, the mean value for the lower limit was calculated to be 13.5 mm (95% CI: 12.4 mm–14.7 mm). The results of the sensitivity study indicated that the distances were reduced when examining the Asian population (12.7 mm; 95% CI: 12.6 mm–12.8 mm) vs. the USA population (14.1 mm; 95% CI: 13.6 mm–14.5 mm).

## 4. Discussion

This study provided a comprehensive analysis of the ITSL morphology based on a systematic review of 14 studies, including 995 scapulae in total. This study confirmed the rarity of the structure by estimating the overall prevalence of the ITSL as 5.8 (95% CI: 4.5–7.1), with significant regional variations: 1.9 in the United States, 19.5 in Europe, and 3.7 in Asia (*p* < 0.01). Additionally, studies with larger sample sizes (>50 scapulae) yielded more accurate prevalence estimates. The ITSL was predominantly ligamentous (4.5; 95% CI: 3.5–5.4) compared to membranous (1.2; 95% CI: 0.8–1.6), with an odds ratio of 0.5 (95% CI: 0.3–0.7), favoring the ligamentous type. Morphologically, the ITSL primarily originated from the scapular spine and inserted at the posterior glenoidal neck. The mean length of the ITSL was found to be shorter in Asian populations compared to those in the USA. These findings underscore the morphological diversity of the ITSL and its potential implications for suprascapular nerve entrapment.

### 4.1. Definition of the ITSL

According to the majority of the classical anatomical textbooks [41,42,43], a ligament is a band or cord of connective tissue connecting bony structures. Morphologically, these ligaments can be of various widths, lengths, or densities. From a histological point of view, ligaments consist either of dense collagen fiber bundles or primarily of elastic tissue, which imparts stretchability. The ITSL is believed to be a complex ligament consisting of both of these two forms [31].

As for the various terminology that has been used in order to describe the ITSL, according to Terminologia Anatomica [44], the two more commonly used terms are ITSL and spinoglenoid ligament, which are considered as synonyms. It is worth mentioning that Bektas et al. proposed the term “spinoglenoid septum” to describe a wide, thin connective and loose tissue structure that they had identified instead of the “usual” ITSL [45]. As far as we are concerned, this term (i.e., spinoglenoid septum) has neither been further used nor included in Terminologia Anatomica. For the purposes of this study, although no further information, either morphometrical or histological, was provided in detail, we would include this variation in the membranous type of the ITSL.

### 4.2. ITSL Prevalence

For many decades, the ITSL was considered an anatomical variation, the prevalence and morphology of which has been debatable. The reported prevalence of the ITSL has been documented to vary between 16% and 100% [33]. It could be suggested that this considerable variation observed may be accounted for by the diverse criteria employed in defining the ITSL, and by its variable morphology, either ligamentous, membranous, or both. Won et al. conjectured that the lowest ITSL prevalence reported in the literature is in those studies in which the ITSL is deemed only ligamentous [33]. In this review, we contend that the ossified variant of the ITSL represents a distinct subtype that has also not been accounted for in the determination of the ITSL prevalence.

### 4.3. Clinical Significance

The first studies regarding the anatomy of the ITSL were conducted in order to investigate the potential correlation between the various ITSL morphology and histology and the development of the SSN entrapment [22,27,32]. The first ever report of the SSN entrapment syndrome was published by Kopell and Thompson in 1959, and since then there has been a cascade of reports and studies concerning the etiology and potential anatomical risk factors of the SSN entrapment, either in the suprascapular or in the spinoglenoid ligament [46].

The main mechanisms that lead to SSN entrapment at the spinoglenoid notch include ganglion cysts [47,48] and the formation of a spinoglenoid foramen by an ossified ITSL [21,29,49] or by a hypertrophic ITSL [11,26]. However, there have even been reported cases of SSN entrapment at the spinoglenoid notch due to the existence of enlarged veins [16].

Moreover, according to the dynamic study of Plancher et al., the glenohumeral joint position has an impact on the ITSL [50]. More specifically, their findings supported that internal rotation leads to an increase in the ITSL tension, which in combination with repetitive shoulder movement can cause distal SSN compression and/or trauma [50].

Clarification of the etiology of suprascapular nerve entrapment is necessary in order to guide appropriate treatment.

### 4.4. Various Morphological Classifications of the ITSL

In the aforementioned original studies on the ITSL anatomy and morphometry, various classifications were employed to facilitate the comprehensive description of the researchers’ findings [12,21,22,23,24,25,26,27,28,29,30,31,32,33]. Cummins et al. (26 years ago) proposed, in their cadaveric study, a rudimentary categorization for the ITSL morphology that included 2 main types that “differed from each other only in terms of thickness” [21]. Judi Ide and co-authors in their study (2003) divided the ITSLs into two basic categories: the “ligamentous type” and the “membranous type” [26].

### 4.5. Our Classification of ITSL Subtypes

Considering the clinical importance of the ITSL and its considerable morphological variability, which has been a source of confusion in studies on ITSL anatomy, it was essential to not only summarize the descriptions of these subtypes in this review but also to present a straightforward classification for ease of recall and identification. Thus, we proposed a system of classification similar to those described for the superior transverse scapular ligament [18].

**Figure 4:**
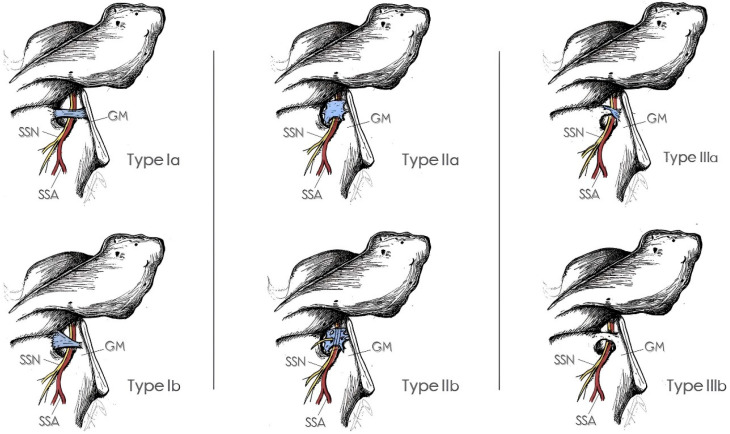
Our proposal for morphological classification of the ITSL (original illustration by I.A.). Type Ia: band-like ligamentous ITSL, Type Ib: fan-shaped ligamentous ITSL, Type IIa: membranous ITSL, Type IIb: thinner and/or ethmoidal membranous ITSL, Type IIIa: a partially ossified ITSL, and Type IIIb: fully ossified ITSL. SSN: suprascapular nerve; SSA: suprascapular artery; GM: posterior glenoidal margin.

According to the classification system we developed, there are three main types of ITSL morphology depending on its texture and composition: Type I refers to the ligamentous ITSL [21,24,25,26,28,33], Type II to the membranous [21,25,26,33], and Type III to an ossified ITSL [12,29,30]. These three main types can be further divided into two subtypes, as follows: Type Ia describes a ligamentous linear (band-like) ITSL, Type Ib a fan-shaped (also referred to as “triangular”) ligamentous ITSL, Type IIa is a wide membranous ITSL, while type IIb is a thinner, ethmoid (perforated) lamina, and finally, Type IIIa refers to a partially ossified ITSL and Type IIIb to a fully ossified one. It is worth noting that Type Ia also includes the hair-like ligament as well as a kind of “hourglass-shaped” ligament (i.e., a quadrangular ligament slightly thinner in its midportion). The types from the aforementioned classification system are depicted in Figure 4.

Considering the extensive variability of the ITSL morphology and the limited number of large anatomical studies conducted on this subject, we posit that the classification system presented here can serve as a valuable tool for anatomists in enhancing their understanding and identifying and, finally, describing this anatomical structure. Furthermore, we believe that through this classification system, clinicians, particularly orthopedics and radiologists, could gain a more comprehensive understanding of the ITSL and be more cognizant of its existence, despite the fact that it does not always resemble a ligamentous structure.

### 4.6. Future Directions

More studies should be conducted regarding the anatomy and morphometry of the ITSL, as well as its prevalence across diverse populations. Prospective cadaveric studies will be crucial for the evaluation of our classification system and its usability. Moreover, radiological studies can serve as valuable tools for estimating the prevalence of the ITSL, especially that of the Type III ITSL. We believe that large radiological studies could also confirm the existence of the Type IIIa ITSL.

### 4.7. Limitations of the Study

In terms of the current systematic review’s limitations, some studies did not clearly disclose the morphological variations of the ITSL in complete detail, but these appeared in the results. In addition, the overall number of studies included was limited. Furthermore, due to the fact that this was a meta-analysis of cadaveric studies, combined with the limited number of studies included and the considerably high heterogeneity of the meta-analysis, it was difficult to extrapolate the results to the general population.

As for the classification system we proposed, it is a system based on macroscopic visual inspection and not on the histological features of each ITSL subtype. Consequently, there is a likelihood that in certain situations, this distinction may not be easily discernible, and potential variations in interpretation among different observers may also occur. In addition, it has to be stated that regarding the Type IIIa of our classification system, no relevant findings of previous original studies exist. However, considering the development of the Type IIIb (which has been reported) as well as the progressive nature of the ossification process, we firmly believe that this subtype does exist, even though it has not been described until now.

## 5. Conclusions

The ITSL is most often of ligamentous nature, and its prevalence seems to vary depending on ethnicity. Its variable morphology can play a significant role in the etiology of the SSN entrapment. More anatomical studies should be conducted in order to unveil the features of the ITSL and describe this unique structure in detail. Hence, it is imperative to reach a consensus on a common ITSL definition as well as standardization of its basic morphological subtypes. We anticipate that the classification of ITSL subtypes we provided here will be both comprehensive and useful to future researchers.

## Figures and Tables

**Table 1:** Main characteristics of the 14 included studies. (*): No mention of the fixation of the cadavers used.

No.	First Author; Year	Country	Type of Study	Scapulae (*n*)	Type of Specimens
1	Cummins; 1998 [21]	USA	Case series	112	Cadavers *
2	Demirhan; 1998 [22]	USA	Case series	23	Fresh frozen cadavers
3	Ide; 1998 [27]	Japan	Case series	40	Cadavers *
4	Ticker; 1998 [32]	USA	Case series	79	Embalmed cadavers
5	Demirkan; 2003 [23]	Turkey	Case series	27	Cadavers *
6	Ide; 2003 [26]	Japan	Case series	115	Cadavers *
7	Plancher; 2005 [31]	USA	Case series	58	Fresh frozen cadavers
8	Duparc; 2010 [24]	France	Case series	30	Embalmed cadavers
9	Kharay; 2010 [29]	India	Case report	1	Dried scapulae
10	Jerosh; 2012 [28]	Germany	Case series	36	Formalin-fixed and Thiel embalmed cadavers
11	Won; 2014 [33]	Korea	Case series	110	Embalmed and fresh cadavers
12	Gandhi; 2016 [25]	India	Case series	90	Cadavers *
13	Kharay; 2016 [30]	India	Case series	268	Dried scapulae
14	Cohn; 2020 [12]	USA	Case report	6	Patients

**Table 2:** Various incidences of the ITSL and its subtypes, as reported in the 14 included studies. (*): Only abstract available.

No.	First Author; Year	Type of Study	Scapulae, *n*	ITSL Prevalence, *n* (%)	Ligamentous, *n* (%)	Membranous, *n* (%)	Mixed, *n* (%)	Ossified, *n* (%)
1	Cummins; 1998 [21]	Case series	112	90 (80.4)	22 (19.6)	68 (60.7)	0 (0)	0 (0)
2	Demirhan; 1998 [22]	Case series	23	14 (60.8)	–	–	–	–
3	Ide; 1998 [27]	Case series *	40	33 (82.5)	–	–	–	–
4	Ticker; 1998 [32]	Case series	79	11 (13.9)	–	–	–	0 (0)
5	Demirkan; 2003 [23]	Case series	27	27 (100)	–	–	–	–
6	Ide; 2003 [26]	Case series	115	94 (81.7)	25 (21.7)	69 (60)	(0)	(0)
7	Plancher; 2005 [31]	Case series	58	58 (100)	–	–	–	–
8	Duparc; 2010 [24]	Case series	30	24 (80)	24 (80)	0 (0)	0 (0)	0 (0)
9	Kharay; 2010 [29]	Case report	1	1 (100)	0 (0)	0 (0)	0 (0)	1
10	Jerosh; 2012 [28]	Case series	36	35 (97.2)	35(97.2)	0 (0)	0 (0)	0 (0)
11	Won; 2014 [33]	Case series	110	73 (66.4)	25 (22.7)	40 (36.4)	8 (7.3)	0 (0)
12	Gandhi; 2016 [25]	Case series *	90	78 (86.7)	25 (27.8)	51 (56.7)	–	–
13	Kharay; 2016 [30]	Case series	268	1 (0.4)	0 (0)	0 (0)	0 (0)	1 (0.4)
14	Cohn; 2020 [12]	Case report	6	6 (100)	0 (0)	0 (0)	0 (0)	6 (100)

**Table 3:** The various incidences of origins and insertions, as well as the different reported lengths of the ITSLs.

No.	First Author; Year	Origin, *n* (%)	Insertion		Length, mm (mean)	
		Scapular Spine	Posterior Glenoidal Neck	Posterior Shoulder Capsule	Upper	Lower
1	Cummins; 1998 [21]	90 (100)	90 (100)	0 (0)	–	–
2	Demirhan; 1998 [22]	14 (100)	0 (0)	14 (0)	16.9	13.7
3	Ticker; 1998 [32]	11 (100)	11 (100)	0 (0)	–	–
4	Demirkan; 2003 [23]	27 (100)	14 (51.8)	5 (18.5)	–	–
5	Plancher; 2005 [31]	58 (100)	58 (100)	0 (0)	15.8	14.2
6	Jerosh; 2012 [28]	–	30 (85.7)	5 (14.3)	–	–
7	Won; 2014 [33]	73 (100)	100 (0)	0 (0)	13.9	12.7
8	Kharay; 2016 [30]	1 (100)	1 (100)	–	–	–
9	Cohn; 2020 [12]	6 (100)	6 (100)	0 (0)	0 (0)	0 (0)

## References

[1] Shin C., Lee S.E., Yu K.H., Chae H.K., Lee K.S. (2010). Spinal root origins and innervations of the suprascapular nerve. Surg. Radiol. Anat..

[2] Basta M., Sanganeria T., Varacallo M. (2024). Anatomy, Shoulder and Upper Limb, Suprascapular Nerve. StatPearls.

[3] Laumonerie P., Blasco L., Tibbo M.E., Bonnevialle N., Labrousse M., Chaynes P., Mansat P. (2019). Sensory innervation of the subacromial bursa by the distal suprascapular nerve: A new description of its anatomic distribution. J. Shoulder Elb. Surg..

[4] Laumonerie P., Dalmas Y., Tibbo M.E., Robert S., Faruch M., Chaynes P., Bonnevialle N., Mansat P. (2020). Sensory innervation of the human shoulder joint: The three bridges to break. J. Shoulder Elb. Surg..

[5] Yang H.J., Gil Y.C., Jin J.D., Ahn S.V., Lee H.Y. (2012). Topographical anatomy of the suprascapular nerve and vessels at the suprascapular notch. Clin. Anat..

[6] Ebraheim N.A., Whitehead J.L., Alla S.R., Moral M.Z., Castillo S., McCollough A.L., Yeasting R.A., Liu J. (2011). The suprascapular nerve and its articular branch to the acromioclavicular joint: An anatomic study. J. Shoulder Elb. Surg..

[7] Brennan P.S.S., Wiseman S.M., Ross A.C. (2020). Supraclavicular and infraclavicular regions. Gray’s Surgical Anatomy.

[8] Labetowicz P., Synder M., Wojciechowski M., Orczyk K., Jezierski H., Topol M., Polguj M. (2017). Protective and Predisposing Morphological Factors in Suprascapular Nerve Entrapment Syndrome: A Fundamental Review Based on Recent Observations. Biomed. Res. Int..

[9] Moen T.C., Babatunde O.M., Hsu S.H., Ahmad C.S., Levine W.N. (2012). Suprascapular neuropathy: What does the literature show?. J. Shoulder Elb. Surg..

[10] Polguj M., Sibinski M., Grzegorzewski A., Grzelak P., Majos A., Topol M. (2013). Variation in morphology of suprascapular notch as a factor of suprascapular nerve entrapment. Int. Orthop..

[11] Aiello I., Serra G., Traina G.C., Tugnoli V. (1982). Entrapment of the suprascapular nerve at the spinoglenoid notch. Ann. Neurol..

[12] Cohn M.R., Cregar W.M., Drager J., Lu Y., Garrigues G.E. (2020). Suprascapular Nerve Entrapment due to an Ossified Spinoglenoid Ligament After Scapular Fracture: A Case Report. JBJS Case Connect..

[13] Kiss G., Komar J. (1990). Suprascapular nerve compression at the spinoglenoid notch. Muscle Nerve.

[14] Neviaser T.J., Ain B.R., Neviaser R.J. (1986). Suprascapular nerve denervation secondary to attenuation by a ganglionic cyst. J. Bone Jt. Surg. Am..

[15] Takagishi K., Saitoh A., Tonegawa M., Ikeda T., Itoman M. (1994). Isolated paralysis of the infraspinatus muscle. J. Bone Jt. Surg. Br..

[16] Carroll K.W., Helms C.A., Otte M.T., Moellken S.M., Fritz R. (2003). Enlarged spinoglenoid notch veins causing suprascapular nerve compression. Skelet. Radiol..

[17] Bayramoglu A., Demiryurek D., Tuccar E., Erbil M., Aldur M.M., Tetik O., Doral M.N. (2003). Variations in anatomy at the suprascapular notch possibly causing suprascapular nerve entrapment: An anatomical study. Knee Surg. Sports Traumatol. Arthrosc..

[18] Polguj M., Jedrzejewski K., Podgorski M., Majos A., Topol M. (2013). A proposal for classification of the superior transverse scapular ligament: Variable morphology and its potential influence on suprascapular nerve entrapment. J. Shoulder Elb. Surg..

[19] Tsikouris G., Antonopoulos I., Vasdeki D., Chrysikos D., Koukakis A., Tsakotos G., Georgakopoulos P., Troupis T. (2021). Morphometry and Contents of the Suprascapular Notch with Potential Clinical Implications: Alpha Cadaveric Study. J. Brachial Plex. Peripher. Nerve Inj..

[20] Tubbs R.S., Nechtman C., D’Antoni A.V., Shoja M.M., Mortazavi M.M., Loukas M., Rozzelle C.J., Spinner R.J. (2013). Ossification of the suprascapular ligament: A risk factor for suprascapular nerve compression?. Int. J. Shoulder Surg..

[21] Cummins C.A., Anderson K., Bowen M., Nuber G., Roth S.I. (1998). Anatomy and histological characteristics of the spinoglenoid ligament. J. Bone Jt. Surg. Am..

[22] Demirhan M., Imhoff A.B., Debski R.E., Patel P.R., Fu F.H., Woo S.L. (1998). The spinoglenoid ligament and its relationship to the suprascapular nerve. J. Shoulder Elb. Surg..

[23] Demirkan A.F., Sargon M.F., Erkula G., Kiter E. (2003). The spinoglenoid ligament: An anatomic study. Clin. Anat..

[24] Duparc F., Coquerel D., Ozeel J., Noyon M., Gerometta A., Michot C. (2010). Anatomical basis of the suprascapular nerve entrapment, and clinical relevance of the supraspinatus fascia. Surg. Radiol. Anat..

[25] Gandhi K.R., Wabale R.N. (2016). The morphology of inferior transverse scapular ligament in human cadavers. J. Anat. Soc. India.

[26] Ide J., Maeda S., Takagi K. (2003). Does the inferior transverse scapular ligament cause distal suprascapular nerve entrapment? An anatomic and morphologic study. J. Shoulder Elb. Surg..

[27] Ide J., Yamaga M., Kitamura T., Maeda S., Kodama K., Takagi K. (1998). Does inferior transverse scapular ligament play a role in suprascapular nerve entrapment neuropathy?—An anatomical study. J. Shoulder Elb. Surg..

[28] Jerosch J., Filler T., Mertens T. (2012). The spinoglenoid ligament—An anatomic study. Z. Orthop. Unf..

[29] Kharay S.S., Sharma A. (2010). The spinoglenoid foramen. Clin. Anat..

[30] Kharay S.S., Sharma A., Singh P. (2016). Unusual morphology of scapulae: Incidence and dimensions of ossified ligaments and supraspinous bony tunnels for clinical consideration. Singap. Med. J..

[31] Plancher K.D., Peterson R.K., Johnston J.C., Luke T.A. (2005). The spinoglenoid ligament. Anatomy, morphology, and histological findings. J. Bone Jt. Surg. Am..

[32] Ticker J.B., Djurasovic M., Strauch R.J., April E.W., Pollock R.G., Flatow E.L., Bigliani L.U. (1998). The incidence of ganglion cysts and other variations in anatomy along the course of the suprascapular nerve. J. Shoulder Elb. Surg..

[33] Won H.J., Won H.S., Oh C.S., Han S.H., Chung I.H., Yoon Y.C. (2014). Morphological study of the inferior transverse scapular ligament. Clin. Anat..

[34] Liberati A., Altman D.G., Tetzlaff J., Mulrow C., Gotzsche P.C., Ioannidis J.P., Clarke M., Devereaux P.J., Kleijnen J., Moher D. (2009). The PRISMA statement for reporting systematic reviews and meta-analyses of studies that evaluate health care interventions: Explanation and elaboration. J. Clin. Epidemiol..

[35] Wohlin C. Guidelines for snowballing in systematic literature studies and a replication in software engineering. Proceedings of the 18th International Conference on Evaluation and Assessment in Software Engineering.

[36] Henry B.M., Tomaszewski K.A., Ramakrishnan P.K., Roy J., Vikse J., Loukas M., Tubbs R.S., Walocha J.A. (2017). Development of the anatomical quality assessment (AQUA) tool for the quality assessment of anatomical studies included in meta-analyses and systematic reviews. Clin. Anat..

[37] Pearce N. (2004). Effect measures in prevalence studies. Environ. Health Perspect..

[38] Munn Z., Moola S., Lisy K., Riitano D., Tufanaru C. (2015). Methodological guidance for systematic reviews of observational epidemiological studies reporting prevalence and cumulative incidence data. Int. J. Evid. Based Healthc..

[39] Higgins J.P.T. (2008). Heterogeneity in meta-analysis should be expected and appropriately quantified. Int. J. Epidemiol..

[40] Deeks J.J., Higgins J.P., Altman D.G., Cochrane Statistical Methods Group (2008). Analysing data and undertaking meta-analyses. Cochrane Handbook for Systematic Reviews of Interventions.

[41] Standring S. (2021). Functional anatomy of the musculosceletal system. Gray’s Anatomy: The Anatomical Basis of Clinical Practice.

[42] Thomas C. (1981). Taber’s Cyclopedic Medical Dictionary.

[43] Wineski L.S., Snell R.S. (2018). Clinical Anatomy by Regions.

[44] Federative Committee on Anatomical Terminology (2019). Terminologia Anatomica: International Anatomical Terminology.

[45] Bektas U., Ay S., Yilmaz C., Tekdemir I., Elhan A. (2003). Spinoglenoid septum: A new anatomic finding. J. Shoulder Elb. Surg..

[46] Kopell H.P., Thompson W.A. (1959). Pain and the frozen shoulder. Surg. Gynecol. Obstet..

[47] Fehrman D.A., Orwin J.F., Jennings R.M. (1995). Suprascapular nerve entrapment by ganglion cysts: A report of six cases with arthroscopic findings and review of the literature. Arthroscopy.

[48] Rachbauer F., Sterzinger W., Frischhut B. (1996). Suprascapular nerve entrapment at the spinoglenoid notch caused by a ganglion cyst. J. Shoulder Elb. Surg..

[49] Demaio M., Drez D., Mullins R.C. (1991). The inferior transverse scapular ligament as a possible cause of entrapment neuropathy of the nerve to the infraspinatus. A brief note. J. Bone Jt. Surg. Am..

[50] Plancher K.D., Luke T.A., Peterson R.K., Yacoubian S.V. (2007). Posterior shoulder pain: A dynamic study of the spinoglenoid ligament and treatment with arthroscopic release of the scapular tunnel. Arthroscopy.

